# Effect of Application of Fe-Glycinate Chelate in Diet for Broiler Chickens in an Amount Covering 50 or 25% of the Requirement on Physical, Morphometric and Strength Parameters of Tibia Bones

**DOI:** 10.1007/s12011-017-1171-3

**Published:** 2017-11-09

**Authors:** Katarzyna Kwiatkowska, Anna Winiarska-Mieczan, Małgorzata Kwiecień

**Affiliations:** Institute of Animal Nutrition and Bromatology, University of Life Sciences in Lublin, Akademicka 13, 20-950 Lublin, Poland

**Keywords:** Fe-glycinate chelate, Fe sulphate, Lovered level, Tibia bone quality, Mineral composition, Broiler chickens

## Abstract

The purpose of the work was to check whether the application of Fe-glycinate chelate in mixtures fed to poultry in an amount covering 50 or 25% of the requirement would decrease the physical, morphometric and strength parameters of tibia bones in male Ross-308 broiler chickens in comparison to groups receiving Fe in an amount covering 100% of the requirement in the form of glycinate chelate or sulphate. It was found that the results for chickens from groups receiving Fe chelate covering 50 or 25% of the requirement were generally not lower than in the sulphate group and were higher than in the group receiving Fe in the amount covering 100% of the requirement. The presented results indicate that the standard requirement for Fe (40 mg kg^−1^ feed) as recommended by producers of Ross chickens may be too high if glycinate chelate is the source of Fe. This can be connected with the higher bioavailability of Fe from organic compounds in comparison to inorganic compounds.

## Introduction

For more than 50 years, intense genetic selection has aimed to increase the rate of weight gain in broiler chickens [1]. Over the past 30 years, the slaughter weight of broiler chickens increased twice. At the same time, the rearing period was reduced by half. However, the intensification of rearing results in the unbalanced growth of anatomical parts of the birds’ bodies, which leads to numerous irregularities, including anomalies of the skeleton, and in particular leg bones, such as deformations, infections and osteoporosis as well as tibial dyschondroplasia [2, 3]. These pathologies are due to the fact that too intense a growth rate prevents the full maturity of the skeleton, which means that the legs are not capable of supporting the heavy-weight broilers’ body. The strength of leg bones is not only genetically determined but it also depends on the sex, age, health condition and nutrition. Therefore, studies are carried out to increase the strength of the broilers’ leg bones.

The correct development of the birds’ bones and their proper functions are affected by multiple environment and nutrition-related factors, including the first place adequate levels of assimilable minerals in the feed mixture [4]. Most often, these minerals are calcium, phosphorus, magnesium and copper. An unappreciated mineral having a significant influence on the bone structure is iron (Fe). In vitro studies showed that with excess Fe the activity of human osteoblasts is inhibited. A moderate Fe deficiency stimulated the activity of osteoblasts, whereas very low levels of Fe inhibited osteogenesis [5]. Iron plays a significant role in bone-forming processes as a co-factor for enzymes involved in the synthesis of collagen and metabolism of vitamin D [6]. Animals receiving low doses of Fe in feed showed poorer bone mineralization and reduced bone strength, weaker bone structure and an increase in resorption markers [6]. However, most data concerning the relationship between bone quality and Fe refers to the excess of this mineral. Studies involving laboratory animals and observation of humans demonstrated a relationship between excessive Fe accumulation in the body as a result of medical conditions disturbing the metabolism of this element (haemochromatosis, thalassemia) and osteoporosis as well as increased susceptibility to bone fracture [7, 8]. Guggenbuhl et al. [9] suggested a relationship between excessive accumulation of Fe in the liver and bones of mice and a reduced osteoclasts count. Tsay et al. [7] noticed anomalies in the cortical bone of mice that were related to considerable Fe overload in bones. In addition, the above-named authors found that the bone weight of mice receiving excessive amounts of Fe was low, which was probably a result of changes in the bone microstructure and simultaneously increased oxidative stress (induced by Fe) during which the increase in the level of reactive forms of oxygen was accompanied by a growth in the level of proosteoclastogenic cytokines and interculin-6 in blood plasma.

Available studies show that both a deficiency and excessive supply of Fe have a negative effect on bones, which in the case of slaughter animals can be connected with improper Fe balancing. Iron available in feed is characterized by a low level of assimilation. However, mineral additives can contain Fe with high bioavailability. Recently, a lot of attention has been paid to mineral chelates. It has been demonstrated that Fe applied in such a form is characterized by better assimilability [10, 11]. However, high bioavailability of Fe from chelates can contribute to excessive supply of Fe, and in turn, increased accumulation of Fe in tissues (including bones) and, as a consequence, can lead to reduced bone strength. In turn, in such a case, Fe deficiency can be caused by low Fe supply, which can be a consequence of too radical assessment of the bioavailability of Fe from chelates. The purpose of the work was to check whether the application of Fe-glycinate chelate in mixtures fed to poultry in an amount covering 50 or 25% of the requirement would decrease the physical, morphometric and strength parameters of tibia bones in male Ross-308 broiler chickens in comparison to groups receiving Fe in an amount covering 100% of the requirement in the form of glycinate chelate or sulphate.

## Material and Methods

### Chickens and Experimental Design

All procedures used during the research were approved by the 2nd Local Ethics Committee for Animal Testing at the University of Life Sciences in Lublin (No. 37/2011 of 17 May 2011). The experiment made use of 200 1-day-old male Ross-308 chickens split into 4 equipotent experimental groups: Fe-sulphate-40, Fe-Gly-40, Fe-Gly-20 and Fe-Gly-10, as presented in Table 1. The broilers were placed in cages, 10 birds in each, in a room with controlled temperature conditions: initially 32 °C, then over 4 weeks lowered to 22 °C. Two experimental factors were used: (1) the form of Fe (inorganic—sulphate and organic—glycinate chelate) and (2) different levels of Fe administered in an organic form (40 mg, 20 mg or 10 mg kg^−1^ feed). The requirement of Fe was determined on the basis of recommendations of producers of Ross 308 broiler chickens [12]. In the Fe-sulphate-40 group, the birds received Fe sulphate; the group was considered as a control one because iron sulphate is a standard feed additive used in the nutrition of broilers in Poland. In experimental groups, birds were administered Fe-glycinate chelate (Table 1). Wheat, corn and soybean meal-based feed mixture was optimised according to NRC standards [13] (Table 2). The birds were weighed on the 1st, 10th, 35th and 42nd day of the experiment. The production performance (feed intake, feed conversion per 1 kg of weight gain, percentage share of the breast and thigh muscles in the carcass, mortality rate) as well as biochemical and morphological blood parameters obtained in this experiment and the accurate composition of feed mixtures are described elsewhere [14]. The studied parameters recorded for broilers receiving 40, 20 or 10 mg of Fe-glycinate chelate did not significantly differ from those of chickens receiving iron sulphate. Results, published in another work [15], concerning the chemical composition of thigh meat also showed no significant effect of the applied experimental factors. On the last, 42nd, day of the experiment the animals were deprived of access to feed 10 h before slaughter but had continuous access to water. The birds were decapitated in compliance with procedures described in Council Regulation (EC) No. 1099/2009 of 24 September 2009. The slaughter was followed by a simplified dissection analysis of 10 broilers from each group [16] during which right-leg tibia of chicks were prepared.

**Table 1:** Experimental design

	Feeding groups			
	Fe-suphate-40	Fe-Gly-40	Fe-Gly-20	Fe-Gly-10
Starter (1–21 days)	Standard mixture^a^ (contained 40 mg Fe kg^−1^ at the form of FeSO_4_)^b^	Standard mixture (contained 40 mg Fe kg^−1^ at the form of glycine chelate)^b^	Standard mixture (contained 20 mg Fe kg^−1^ at the form of glycine chelate)^c^	Standard mixture (contained 10 mg Fe kg^−1^ at the form of glycine chelate)^d^
Grower (22–35 days)	Standard mixture (contained 40 mg Fe kg^−1^ at the form of FeSO_4_)^b^	Standard mixture (contained 40 mg Fe kg^−1^ at the form of glycine chelate)^b^	Standard mixture (contained 20 mg Fe kg^−1^ at the form of glycine chelate)^c^	Standard mixture (contained 10 mg Fe kg^−1^ at the form of glycine chelate)^d^
Finisher (36–42 days)	Standard mixture (contained 40 mg Fe kg^−1^ at the form of FeSO_4_)^b^	Standard mixture (contained 40 mg Fe kg^−1^ at the form of glycine chelate)^b^	Standard mixture (contained 20 mg Fe kg^−1^ at the form of glycine chelate)^c^	Standard mixture (contained 10 mg Fe kg^−1^ at the form of glycine chelate)^d^
Access to feed and water	Free	Free	Free	Free
Number of chickens in the experiments	50^e^	50^e^	50^e^	50^e^
Number of chickens for dissection	10	10	10	10

^a^Composition of the standard mixtures: maize, wheat, soybean meal 46%, soybean oil, monocalcium phosphate, limestone, sodium bicarbonate, NaCl, vitamin-mineral premix, fat-protein concentrate, DL-methionine 99%, l-lysine HCl, l-threonine 99%

^b^At 100% recommendation levels for Ross broiler chicks [12]

^c^At 50% recommendation levels for Ross broiler chicks [12]

^d^At 25% recommendation levels for Ross broiler chicks [12]

^e^5 cages × 10 birds in each

**Table 2:** Nutritive value of standard mixtures *

	Starter (1–21 days)	Grower (22–35 days)	Finisher (36–42 days)
Values calculated			
Energy (MJ kg^−1^)	12.71	13.12	13.22
Crude protein, %	20.21	18.21	18.13
Crude fiber, %	3.060	2.991	2.992
Crude fat, %	4.660	6.081	6.434
Lysine, %	1.293	1.133	1.090
Met + Cys, %	0.934	0.831	0.812
Values determined			
Ca total, %	0.883	0.781	0.754
P total, %	0.656	0.654	0.634
P available, %	0.422	0.409	0.389
Ca total/P available	2.121	1.900	1.921
Cu, mg kg-1	14.02	14.12	13.80
Zn, mg kg^−1^	99.67	98.54	98.53
Fe, mg kg^−1^			
Fe-suphate-40	113.6	109.8	106.7
Fe-Gly-40	110.3	107.3	104.6
Fe-Gly-20	90.32	89.82	85.43
Fe-Gly-10	83.44	79.80	76.91

Composition of the basal mixtures: maize, wheat, soybean meal 46%, soybean oil, monocalcium phosphate, limestone, sodium bicarbonate, NaCl, vitamin-mineral premix, fat-protein concentrate, DL-methionine 99%, l-lysine HCl, l-threonine 99%

### Bone Measurements

Bone samples were cleared off soft tissue using mechanical methods. The bones were weighed using digital scales. Next, their lengths were measured using electronic callipers (accuracy up to 0.001 mm), always in the same position during the measurement, and the circumference was measured along ½ of the bone length. Based on the resulting parameters, the bone density ratio was determined. It was calculated as bone weight (mg) to length (mm). Afterwards, each bone was wrapped in a piece of gauze saturated with an isotonic saline solution and stored in identified film bags at a temperature of − 25 °C until further analyses.

### Mechanical Properties of Bones

Prior to analyses, the tibia bones were thawed for 3 h at room temperature. Their mechanical properties were measured by means of a 3-point bend test using a Zwick Z010 testing apparatus (Zwick GmbH & Co KG). A measuring head (Zwick GmbH & Co KG) with the operating range up to 10 kN was used at a fixed speed of 10 mm min^−1^. The bones for testing were placed on supports spaced at 40% of bone length. The results were analysed using TestXpert II 3.1 software (Zwick GmbH & Company KG). The apparatus was used to measure the maximum ultimate strength and the maximum elastic strength. Based on these measurements the yielding deformation, bending point resistance, load-to-deformation ratio, bending point resistance, Young’s modulus and bone density index were calculated as described in the works by Kwiecień et al. [17, 18] and Kwiatkowska et al. [19].

### Geometric and Cortical Properties of Bones

Geometric and cortical features of tibia were calculated based on the measurements of the outer and inner horizontal and vertical diameter of the cross section of the bone shaft in the place of fracture. The measurements were made using electronic callipers (accuracy up to 0.001 mm). The following cortical bone parameters were calculated: second moment of inertia of the cross section in relation to the horizontal axis, cross-sectional area and mean relative wall thickness, as described in other works [17–19]. Other cortical bone parameters that were calculated included: the thickness of cortical layer, cortical surface, cortical index and cortical surface index, as described in the work by Kwiecień et al. [17, 18].

### Chemical Analyses

Prior to chemical analyses, the bones were degreased with a mixture of diethyl ether and ethanol, dried to constant weight and subjected to dry mineralization in a muffle furnace at a temperature of 550 °C [20] using hydrogen peroxide as an oxidant. The ashed bone samples were dissolved in 10 ml of 1 M HNO_3_ solution. The content of Ca, Mg, Cu, Fe and Zn in ashed bone samples was determined by means of Atomic Absorption Spectrometry in a Unicam 939/959 apparatus (Shimadzu, Tokyo, Japan) using Merck’s reference standards (Germany) [18, 21]. The total P content was determined by colorimetric methods according to PN-76/R-64781 in a Helios α-UV-VIS apparatus (Spectronic Unicam, Leeds, UK) at *λ* = 430 nm, using molybdenum and vanadium as the reagent (NH_4_VO_3_, (NH_4_)_6_Mo_7_O_24_·H_2_O, H_2_O) [21].

## Results

Table 3 presents the physical, mechanical, geometric and cortical parameters of tibia bones in broiler chickens. The highest bone weight was recorded in the group Fe-Gly-20, where this value was significantly higher in comparison to the Fe-sulphate-40 group (23.45 vs 21.91 g). However, no difference (*P* < 0.05) was found between values recorded in the groups Fe-Gly-40, Fe-Gly-20 and Fe-Gly-10. The experimental factors were found to have a significant influence on certain mechanical parameters of tibia. In comparison to the Fe-sulphate-40 group, higher (*P* < 0.05) parameters were obtained for the maximum elastic strength towards bone weight in the group Fe-Gly-20 and for the maximum elastic strength and yielding deformation in the groups Fe-Gly-20 and Fe-Gly-10. However, statistically confirmed lower effects than in the Fe-sulphate-40 group were achieved in the group Fe-Gly-40 for the following parameters: maximum elastic strength towards bone and body weight, maximum elastic strength and yielding deformation. The amount of Fe administered as chelate had a significant influence on the analyzed parameters. It was found that the lowest values of the maximum elastic strength towards bone and body weight, maximum elastic strength and yielding deformation were noted down in chickens from the group Fe-Gly-40 in comparison to the groups Fe-Gly-20 and Fe-Gly-10. No significant differences were recorded between the values of these parameters in the groups Fe-Gly-20 and Fe-Gly-10. No significant influence of the experimental factors on the geometric and cortical parameters of tibia was noted (Table 3).

**Table 3:** Physical, morphometric and strength parameters of tibia (mean values)

	Fe-sulphate-40	Fe-Gly-40	Fe-Gly-20	Fe-Gly-10	SEM	Influence of			
						Source^A^		Dose^B^	
						*P* value	Sign	*P* value	Sign
Physical parameters									
Weight, g	21.91^b^	22.21^ab^	23.45^a^	23.04^ab^	0.473	0.004	*	0.118	NS
Weight, g per 100 g of body weight	0.99	0.98	1.02	1.00	0.028	0.812	NS	0.326	NS
Length, mm	110.5	112.1	110.4	110.4	1.108	0.066	NS	0.159	NS
Perimeter, mm	30.38	29.63	29.38	29.00	0.862	0.065	NS	0.236	NS
Mechanical parameters									
Maximum force moment, N mm	268.8	273.9	263.6	282.1	12.99	0.132	NS	0.117	NS
Maximum elastic strength towards bone weight, N mm g^−1^	7.101^b^	6.320^c^	7.284^a^	7.192^ab^	0.291	0.014	*	0.026	*
Maximum elastic strength towards body weight, N mm 1000 kg^−1^	70.17^a^	61.88^b^	73.82^a^	71.97^a^	2.923	0.046	*	0.012	*
Maximum ultimate strength towards bone weight, N mm g^−1^	12.28	12.40	12.35	12.22	0.634	0.358	NS	0.152	NS
Maximum ultimate strength towards body weight, N mm·1000 kg^−1^	121.7	121.0	114.8	123.0	6.465	0.075	NS	0.142	NS
Yielding load, N mm	155.7^b^	140.5^c^	169.6^a^	165.2^a^	5.932	0.025	*	0.009	*
Yielding deformation, mm	1.353^b^	1.305^c^	1.385^a^	1.396^a^	0.056	0.003	*	0.002	*
Load-to-deformation ratio, N mm mm^−1^	115.5	110.2	123.5	119.7	5.952	0.400	NS	0.136	NS
Bending point resistance, N mm mm^−2^	16.42	17.33	16.96	17.11	1.038	0.078	NS	0.452	NS
Young’s modulus, N m^−2^	2.153	2.116	1.880	2.025	0.173	0.977	NS	0.148	NS
Bone density index, mg mm^−1^	198.4	198.0	212.5	208.6	4.147	0.069	NS	0.199	NS
Geometrical parameters									
Second moment of inertia of the cross section in relation to the horizontal axis, mm^4^	100.3	101.6	104.5	103.9	5.979	0.325	NS	0.394	NS
Cross-sectional area, mm^2^	13.70	13.58	12.69	13.89	1.118	0.942	NS	0.852	NS
Mean relative wall thickness	0.264	0.260	0.224	0.264	0.028	0.948	NS	0.362	NS
Thickness of cortical layer, mm	2.100	2.225	2.100	2.225	0.173	0.605	NS	0.895	NS
Cortical parameters									
Surface, mm^2^	20.84	21.03	20.03	20.96	2.244	0.808	NS	0.925	NS
Index, %	7.360	7.314	7.421	7.253	0.167	0.091	NS	0.335	NS
Surface index, %	69.74	66.44	72.89	66.51	2.520	0.359	NS	0.289	NS

*NS* not significant at *P > 0.05*

^A^Iron sulphate vs Fe glycine chelate

^B^Different levels of Fe administered in an organic form (40 mg, 20 mg or 10 mg kg^−1^ feed)

^a,b,c^Means with different superscripts in the same column differ at *P < 0.05*

*Significant at *P ≤ 0.05*

A significant effect of the experimental factors on the mineral composition of tibia was noted (Table 4). In comparison to the Fe-sulphate-40 group, the content of Mg was higher (*P* < 0.05) in the group Fe-Gly-10, the content of Zn was higher in the groups Fe-Gly-20 and Fe-Gly-10 and the content of Cu was higher in the group Fe-Gly-40. It is significant that in the group Fe-Gly-10 the content of Fe was significantly lower than in all other groups, whereas in the group Fe-Gly-40 the content of Fe was significantly higher than in other groups receiving chelates. The amount of Fe administered as chelate had a significant influence on the content of certain minerals in the analyzed bones. In the group Fe-Gly-10 significantly more Mg was recorded compared to the group Fe-Gly-20 (7.66 vs. 7.16 mg kg^−1^). At the same time, it was determined that the relationship between the content of Fe in bones and the supply of Fe chelate can be recorded as Fe-Gly-40 > Fe-Gly-20 > Fe-Gly-10.

**Table 4:** The mineral composition of crude ash of tibia

	Fe-sulphate-40	Fe-Gly-40	Fe-Gly-20	Fe-Gly-10	SEM	Influence of			
						Source^A^		Dose^B^	
						*P* value	Sign	*P* value	Sign
Crude ash, %	17.51^ab^	16.32^b^	18.23^a^	17.32^ab^	0.488	0.454	NS	0.039	*
Ca, g kg^−1^	264.4	275.0	271.3	274.1	2.924	0.078	NS	0.335	NS
P, g kg^−1^	176.2	180.3	174.4	171.7	2.872	0.422	NS	0.039	NS
Mg, g kg^−1^	7.019^b^	7.310^ab^	7.163^b^	7.663^a^	0.189	0.015	*	0.040	*
Zn, mg kg^−1^	492.9^b^	523.9^ab^	556.7^a^	563.5^a^	16.28	< 0.001	*	0.008	NS
Cu, mg kg^−1^	5.647^b^	6.394^a^	5.869^ab^	5.927^ab^	0.491	0.042	*	0.780	NS
Fe, mg kg^−1^	449.4^ab^	460.0^a^	421.4^b^	369.5^c^	23.08	0.003	*	0.024	*

*NS* not significant at *P > 0.05*

^A^Iron sulphate vs Fe glycine chelate

^B^Different levels of Fe administered in an organic form (40 mg, 20 mg or 10 mg kg^−1^ feed)

^a,b,c^Means with different superscripts in the same column differ at *P < 0.05*

*Significant at *P ≤ 0.05*

## Discussion

The presented studies determined that the use of Fe chelate in the nutrition of broiler chickens significantly increased some of the mechanical parameters of tibia bones compared to chickens fed Fe sulphate. It referred to the maximum elastic strength towards bone and body weight, maximum elastic strength and yielding deformation. However, it should be emphasized that these parameters were increased only in groups receiving Fe chelate in amounts covering 50 and/or 25% of the chicks’ requirement of this element [12]. Based on the bone strength analysis, it can be stated that the tibia of chickens receiving reduced doses of Fe was more elastic. The analysis of both experimental factors (mineral source of Fe—sulphate or organic source—chelate; and chelate level) in the presented studies showed that the lowest (*P* < 0.05) mechanical bone parameters were recorded in chickens receiving 40 mg of Fe chelate. It was an amount covering 100% requirement of that element as recommended by producers of Ross 380 broiler chickens [12]. The results of studies carried out by other authors [7–9] testify to a negative influence of excessive amounts of Fe on bone structure and functions. Therefore, it can be stated that the amount of Fe used in the presented experiment (40 mg Fe kg^−1^ feed) in a dose for broiler chickens is too high. In particular, considering that the results for chickens from other groups receiving Fe chelate covering 50 or 25% of the requirement generally were not lower than in the sulphate group and were higher than in the group receiving Fe in the amount covering 100% of the requirement. The results can be a consequence of higher bioavailability of Fe supplied as an organic compound [10, 11]. The thesis about the excess of Fe in the diet is supported by the results of blood analysis of broilers from the presented study published elsewhere [14]. According to them, the blood of birds receiving Fe chelate contained significantly more Fe than that of birds receiving Fe sulphate. Also, results obtained in the presented studies clearly indicate that the content of Fe is significantly higher in the bones of chickens receiving Fe chelate in the amount covering 100% of the requirement in comparison to chickens receiving Fe sulphate. In turn, the results of the study concerning the content of Fe in the femoral bones of broiler chickens receiving Fe-glycinate chelate, presented elsewhere, did not show any effect of this form of iron on the content of this element in bones [21].

The mechanism behind chelates is not completely clear. However, it is believed that elements deriving from mineral and organic compounds can be absorbed in an unchanged form by the intestinal mucous membrane through the amino acid transport system and are thus better assimilated by the organism [22–25]. It is also believed that higher bioavailability of minerals from chelates is due to the fact that they are bound with the amino acid, and thus do not form inassimilable complexes with anti-nutrients such as phytates or non-starch polysaccharides [26, 27]. What is more, it has been demonstrated that iron-glycinate chelate is better absorbed and used than when it derives from other amino acid chelates [10]. In addition, it was found that the stability and availability of intestinal glycinate chelates was 25% higher than in chelates based on lysine or methionine [11]. This means that if broilers are fed recommended doses of Fe-glycinate chelate, the birds can receive excessive amounts of this mineral, which affects bone structure.

Iron homeostasis is maintained only thanks to absorption control since the body has no other mechanism capable of adapting the excretion of this element to current needs. The hormone reducing the absorption of iron by enterocytes and inhibiting the release of iron by macrophages is hepcidin [28]. The mechanism behind hepcidin comprises binding with the ferroportin present on the surface of macrophages, hepatocytes and enterocytes. Low concentration of hepcidin in blood leads to increased activity of ferroportin, which is manifested in an increased level of iron in blood, high degree of transferrin saturation and excessive build-up of iron in the liver and other tissues, including bones [29, 30]. Studies involving mice demonstrated that excessive accumulation of Fe in the body leads to increased resorption in bones and to oxidative stress that cause microstructural changes in bones, which in turn contributes to bone weight reduction [7]. In the presented studies no significant reduction in the weight of tibia of chickens receiving Fe chelate was recorded, which can suggest that although 40 mg Fe did reduce some mechanical parameters of the bone, the amount was not sufficient to radically deteriorate the quality of tibia bones. However, one needs to be careful using large amounts of Fe-glycinate chelate. The results of studies concerning femurs, presented elsewhere [21], did not show a negative effect of using Fe-glycinate chelate on the physical, mechanical and geometric characteristics of the bones. However, it turned out that with a dose of Fe in such a form amounting to 40 mg kg^−1^ feed the results did not significantly differ from those obtained with a dose of Fe amounting to 20 mg kg^−1^ feed. The lack of statistically significant differences in broilers receiving Fe in the form of sulphate or glycinate chelate both as regards the content of minerals and most of the analyzed morphometric and strength properties of femurs [21] and tibia [presented study] points to a positive effect of organic Fe on the structure and functions of leg bones. This is supported by the lack of significant changes in alkaline phosphatase activity in the blood of broilers receiving Fe-glycinate chelate compared to those receiving Fe sulphate [14] as well as the lack of variance in Zn level in blood [14] and Zn and Mg in the femoral bone [21]. In addition, few differences in the analyzed parameters found in the tibia of broilers receiving sulphate or chelate referred to improvement in those parameters, which is a particular advantage because tibia bones growing faster and being subject to higher loads due to the fact that they support the whole body weight of a bird, are more exposed to deformations (mostly dyschondroplasia) than femoral bones [2, 3, 31, 32]. Attention must be paid to the significant increase in the content of Mg and Zn in the tibia of groups supplemented with Fe corresponding to 50 and 25% of the recommended requirement in comparison to those receiving Fe covering 100% of the requirement. It is important for tibia that are susceptible to damage and deformation because Mg and Zn have a stimulating effect on osteoblasts and an inhibitory effect on osteoclasts [33, 34].

To sum up, the presented results indicate that standard requirement of Fe (40 mg kg^−1^ feed) as recommended by producers of Ross chickens may be too high if the source of Fe is glycinate chelate. It can be connected with higher bioavailability of Fe from organic compounds in comparison to inorganic compounds. The analyses of production performance and slaughter parameters of chickens described elsewhere [14], physical, mechanical and geometric characteristics of femoral bones [21] and meat quality [15] carried out by our team, as well as results presented in this work clearly indicate that if Fe-glycinate chelate is used, the sufficient amount is that covering 50% (20 mg Fe kg^−1^ feed) or even 25% (10 mg Fe kg^−1^ feed).
